# Long-term microglial phase-specific dynamics during single vessel occlusion and recanalization

**DOI:** 10.1038/s42003-022-03784-0

**Published:** 2022-08-19

**Authors:** Xiaoke Xie, Xuanting Liu, Jiazhu Zhu, Yongxian Xu, Xiaojing Li, Yameng Zheng, Shangyue Gong, Xiao Xiao, Yiwen Chen, Jianmin Zhang, Wei Gong, Ke Si

**Affiliations:** 1 Department of Psychiatry, First Affiliated Hospital, Zhejiang University School of Medicine, Hangzhou, 310003 China; 2 College of Biomedical Engineering and Instrument Science, Zhejiang University, Hangzhou, 310027 China; 3 MOE Frontier Science Center for Brain Science & Brain-Machine Integration, NHC and CAMS Key Laboratory of Medical Neurobiology, School of Brain Science and Brain Medicine, Zhejiang University, Hangzhou, 310058 China; 4 State Key Laboratory of Modern Optical Instrumentation, College of Optical Science and Engineering, Zhejiang University, Hangzhou, 310027 China; 5 Center for Neuroscience and Department of Neurobiology of the Second Affiliated Hospital, State Key laboratory of Modern Optical Instrumentation, Zhejiang University School of Medicine, Hangzhou, 310009 China

**Keywords:** Neuro-vascular interactions, Stroke

## Abstract

Vascular occlusion leading to brain dysfunctions is usually considered evoking microglia-induced inflammation response. However, it remains unclear how microglia interact with blood vessels in the development of vascular occlusion-related brain disorders. Here, we illuminate long-term spatiotemporal dynamics of microglia during single vessel occlusion and recanalization. Microglia display remarkable response characteristics in different phases, including acute reaction, rapid diffusion, transition and chronic effect. Fibrinogen-induced microglial cluster promotes major histocompatibility complex II (MHCII) expression. Microglial soma represents a unique filament-shape migration and has slower motility compared to the immediate reaction of processes to occlusion. We capture proliferative microglia redistribute territory. Microglial cluster resolves gradually and microglia recover to resting state both in the morphology and function in the chronic effect phase. Therefore, our study offers a comprehensive analysis of spatiotemporal dynamics of microglia and potential mechanisms to both vessel occlusion and recanalization. Microglial phase-specific response suggests the morphological feature-oriented phased intervention would be an attractive option for vascular occlusion-related diseases treatments.

## Abstract (web summary)

The spatiotemporal dynamics of the microglial inflammatory response to single vessel occlusion and recanalization are analysed, revealing four different response phases.

## Introduction

Vascular occlusion, as one of the primary causes of disability, is implicated in multiple disease factors. Diabetes impairs vascular endothelium, makes vessel walls thicker and greatly increases risk of ischemic stroke;^1^ Amyloid-beta reduces cerebral blood flow and vascular abnormalities are often present in Alzheimer’s disease patients^2–4^. Vascular occlusion leads to neural deficits, infarction and cognitive dysfunctions^5–7^. For example, microinfarct column cause depression of neuronal activity in the barrel cortex; Single microinfarcts impair cognitive decision in a macrovibrissa based gap-crossing task. Vascular occlusion evokes inflammation response in the brain^8–10^. However, the mechanism of the interaction between the vascular occlusion and inflammation response is not fully elucidated. As the main effectors of the innate inflammatory response in the central nervous system (CNS), microglia have been extensively studied in vascular occlusion-related diseases^6,11–17^.

Microglia are regarded as one of the most important immune defense lines in the CNS^18–21^. They are highly active in the intact brain, constantly survey their territory as well as interact with other cortical elements^22–24^. Microglia processes extend toward the excitotoxicity neuron after cerebral ischemia to decrease excitotoxic injury in a P2Y12 receptor-dependent manner through altered somatic junctions^12,25^. In addition to microglia-neuron interaction^26–29^, the correlation between microglia and blood vessels has drawn increasing attention in the pathological progression of occluded vascular dysfunctions^14,30–32^. Blood vessels bring adequate oxygen and glucose delivery, which are required for microglia function and viability. Using the photothrombotic approach, researchers found that the activity of microglia is affected by the blood flow near its cell body, rather than the microenvironment around distal microglial processes^33^. Microglia perform phagocytosis leading to vascular disintegration in the middle cerebral artery occlusion (MCAO) treated mouse^31,34^. However, the traditional vessel occlusion operations, such as photothrombotic and MCAO model, are limited by the dysfunction resulting from multiple vascular occlusions and inherently activated microglia (induced by in vitro slicing procedure). These factors would intervene the morphological analysis of microglia. The functional performance of microglia is closely associated with changes in its morphology and motility, thus the specific morphological structure or displacement could indicate microglial functional activities. The function of microglia in extending processes is impaired with P2Y12 receptors inhibition^11^. Microglia migrate to the contralateral hemisphere through the corpus callosum after cortical microinfarcts in a CX3CR1-dependent manner to affect remote brain regions reorganization^6^. Moreover, microglia form clusters following genetic ablation to achieve repopulation^35^. To explore the function of microglia in neurological diseases, it is necessary to investigate the temporally precise microglia morphological dynamics and underlying mechanisms in diverse blood vessels microenvironment in vivo.

Currently, most study of microglia dynamics properties and transformation mechanisms focus on a certain morphological change of microglia during the acute reaction phase. Microglial rapid chemotactic response is regulated by extracellular adenosine triphosphate (ATP) released from damaged tissue and nearby astrocytes after the laser microlesion in the brain parenchyma^11,36^. Microglia converge on the laser microlesion site, forming a rounded protective barrier to prevent brain damage from further spreading^37^. However, the microglial complicated spatiotemporal morphological transformation characteristics are not well investigated in the long-term scale. Here, we applied laser ablation technique^38^ to occlude single vessels and performed long-term in vivo imaging with two-photon laser scanning microscope (TPLSM) to investigate the response of microglia during both vessel occlusion and recanalization phase. We describe the microglia dynamics with four different phases: acute reaction, rapid diffusion, transition and chronic effect during the long-term imaging process. Immunohistochemistry was performed to reveal potential mechanisms. These findings give insights into the spatiotemporal dynamics of microglia and potential mechanisms during vessel occlusion and recanalization, which might lay the foundation on modulating microglial activities for vascular occlusion-related diseases treatments.

## Results

### Establishment of single vessel occlusion in CX3CR1-GFP mouse

**Fig. 1:**
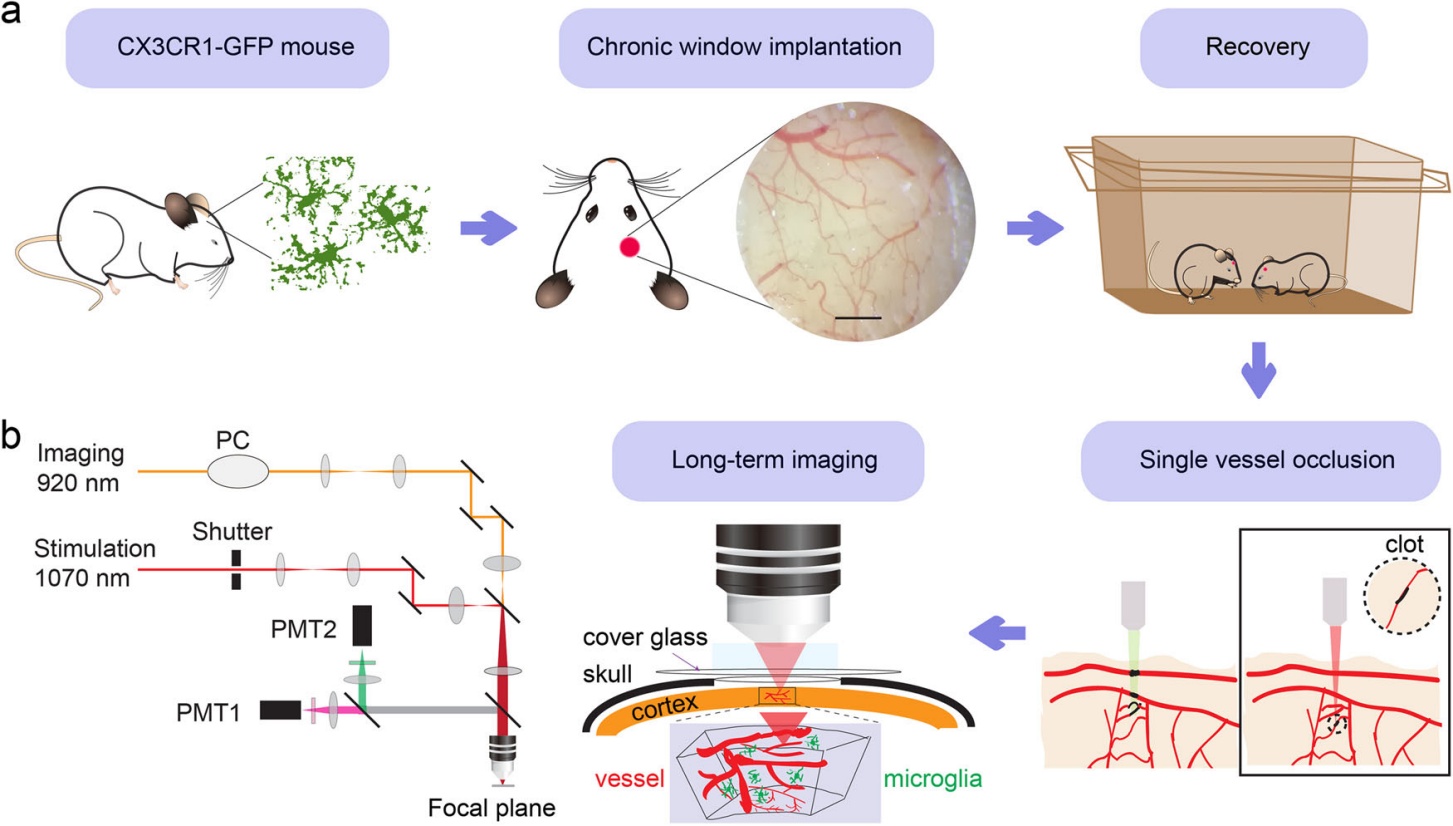
Experimental setup. **a** Flowchart for the experimental procedure. CX3CR1-GFP mice were used for all experiments. After chronic window implantation was accomplished, mice were housed for about 4-weeks recovery from the surgery. Then with two-photon laser stimulation, single vessel occlusion was established (indicated by the dashed line). By comparison, conventional photothrombosis causes all vessels within the light irradiation volume blocked (indicated by the black-colored blood vessels). Next, we performed real-time in vivo imaging of blood vessels and microglia by TPLSM through the specific cranial window. Scale bar, 0.5 mm. **b** Schematic of the two-photon laser scanning microscope modified for laser-induced occlusion and imaging simultaneously. PC Pockels cell, PMT photomultiplier tubes.

We applied laser ablation technique through cranial window to occlude the single vessel to performed long-term in vivo imaging of microglia response (Fig. 1a). As the primary immune effector cells in the brain, microglia are activated and their morphology changes with functional transformations in response to any kind of brain damage or injury. To eliminate surgery-induced effects on the morphology of microglia, we performed long-term imaging after cranial window implantation to record microglia and blood flow state influenced by the surgery. We imaged the morphology of microglia in the same field of view (FOV) for up to one month continuously (Supplementary Fig. 1a). According to the process tree area and the blood flow velocity results (Supplementary Fig. 1b, c), about 4-weeks recovery before the single vessel occlusion is necessary for ruling out the influence factor brought by cranial window implantation. Additionally, increases in imaging quality over time can be observed at day 25, and the morphology of microglia had the appearance of ramified processes (Supplementary Fig. 1a).

Subsequently, we made the model of single vessel occlusion. The vessel diameters varied from 5 μm to 100 μm. We chose the vessel with diameter of ~20 μm and depth of ~80 μm from the pia as the targeted vessel. Insult to the targeted vessel was generated by irradiation of the lumen of the vessel (Fig. 1b). This process provided real-time feedback on the progress of vessel diameter and blood flow velocity changing (Supplementary Fig. 2a–c). When a single vessel is occluded, the clotted site was full of accumulated nonfluorescent blood cells, which was visualized as a dark lump within the vessel (Supplementary Fig. 2b). It is worth noting that the appropriate laser power should be set to avoid hemorrhage by vessel rupture (Supplementary Fig. 3a, b).

### Long-term in vivo imaging of microglia and blood vessel after vessel occlusion

**Fig. 2:**
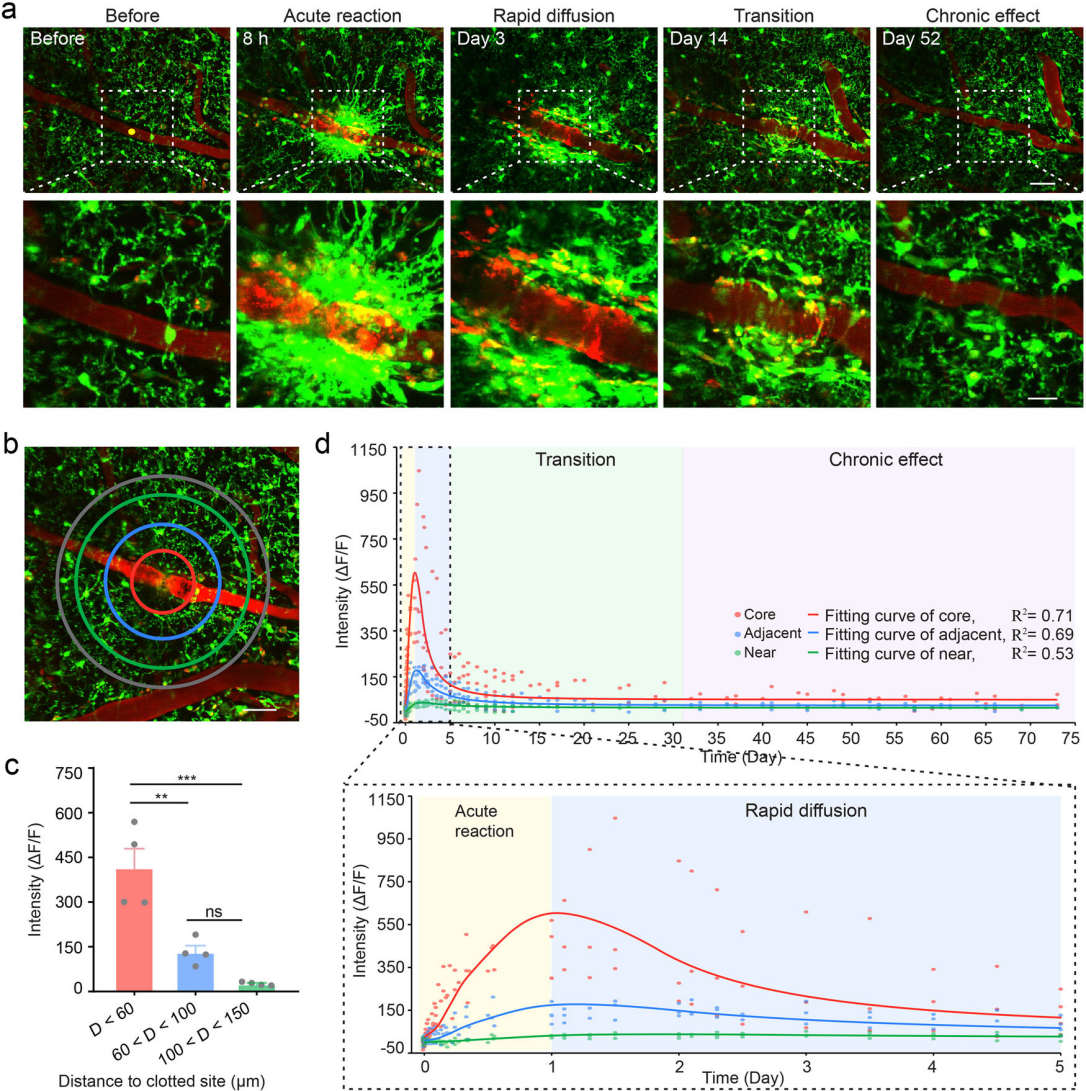
Long-term in vivo imaging after vessel occlusion. **a** Representative maximum intensity projections of 3D z-stack images show microglia (green) and vessels (red) in the normal physiological state (before), acute reaction phase, rapid diffusion phase, transition phase and chronic effect phase. The stacks are 20 μm in depth (2 μm step size). Yellow circle indicates laser irradiation site. White square shows higher magnifications of microglia and vessel morphology. Scale bars, top: 50 μm; bottom: 20 μm. **b** To quantify microglia response toward vessel occlusion, relative fluorescence intensity at different sites is measured from the inner area to the outer area (core, adjacent, near) with different colors. Grey circle area serves as the baseline. Scale bar, 50 μm. **c** Relative intensity quantification of different areas at 24 h after occlusion. D represents distance to the clotted site (*n* = 4 mice, **P < 0.01, ***P < 0.001, ns indicates no significant difference, one-way ANOVA followed by Tukey’s multiple comparison). Error bars, mean and SEM. **d** Scatterplot showing relative intensity quantification as shown in **b**, *n* = 4 mice. Fitting curves show changing tendency of fluorescence intensity over time. Different colors shadow represents different phases. Boxed region indicates magnified view of dotted line.

We imaged the dynamics of microglia caused by laser-induced vascular occlusion for more than 70 days. In this process, microglia experienced morphological transformation from aggregation mass to diffusion individual. According to the dynamic characteristics, we divided the whole pathological process into four phases: acute reaction phase (0–24 h), microglia undergo dramatic morphological changes and aggregate; rapid diffusion phase (day 1-day 5), microglial clusters diffuse rapidly; transition phase (day 5-day 31), microglial cluster exists and individual microglia display ramified; chronic effect phase (>=day 32), microglia recover to resting state (Fig. 2a).

We employed relative fluorescence intensity of occlusion site-centered different areas (core, adjacent, near) to quantify microglia response (Fig. 2b). Results demonstrate that the closer to the occlusion site, the higher the fluorescence intensity of the microglia (Fig. 2c). Beginning with a general polynomial curve fitting of fluorescence intensity changes over time, we found fluorescence intensity changes can be roughly divided into increasing and decreasing periods. To investigate theoretically possible mechanism of microglial phase-specific dynamics, we used S-curve^39,40^ and diffusion-consumption equation^41,42^ to fit our data. In the acute reaction phase, fluorescence intensity shows an S-curve-like increasing tendency. Subsequently, the diffusion-consumption equation was used to fit fluorescence intensity data accompanied by recanalization. According to the fitting curve, results show that fluorescence intensity of core area increased dramatically in the acute reaction phase with the average change rate of fluorescence intensity is 634.18 (*ΔF/F*)/day (Fig. 2d; Supplementary Data 1). According to S-curve theory, the possible mechanism is that occlusion leads to related cytokines release and these cytokines make microglia quickly gather towards the occlusion site. After the surrounding microglial sensors are occupied, more cytokines release cannot increase microglial chemotaxis and the fluorescence intensity reaches the maximum at this time. In the rapid diffusion phase, fluorescence intensity of occlusion core decreased (Fig. 2d). The average change rate of fluorescence intensity of core, adjacent and near area is 119.45, 34.16 and 2.11 (*ΔF/F*)/day, respectively. This suggests that microglial diffusion rate is much slower compared with its rapid aggregation. In the transition phase, the average change rate of fluorescence intensity of core, adjacent and near area is 5.24, 1.69 and 0.41 (*ΔF/F*)/day, respectively. In the chronic effect phase, the average change rate of fluorescence intensity of core, adjacent and near area is 0.41, 0.17 and 0.08 (*ΔF/F*)/day, respectively. These results indicate that the decrease rate of fluorescence intensity slows down over time. Considering the diffusion-consumption mechanism^41^, we speculate that when microglial fluorescence intensity is at a high level and in a rapid decline stage, the spatial spread size of diffusion-effect cytokines is small. On the contrary, diffusion-effect cytokines display a big spatial spread. At the same time, we observed that the blood flow began to recover spontaneously about 1.5 days after occlusion (Supplementary Fig. 4a-c; Supplementary Data 2). Unexpectedly, blood flow velocity has been in a fluctuating state in the following days, accompanied by vascular malformation (Supplementary Fig. 4d). We ruled out surgery-induced vascular malformation by demonstrating that vascular morphology persisted in the area far away from occlusion site (Supplementary Fig. 5).

### Fibrinogen-induced microglial cluster promotes MHCII expression after vessel occlusion

**Fig. 3:**
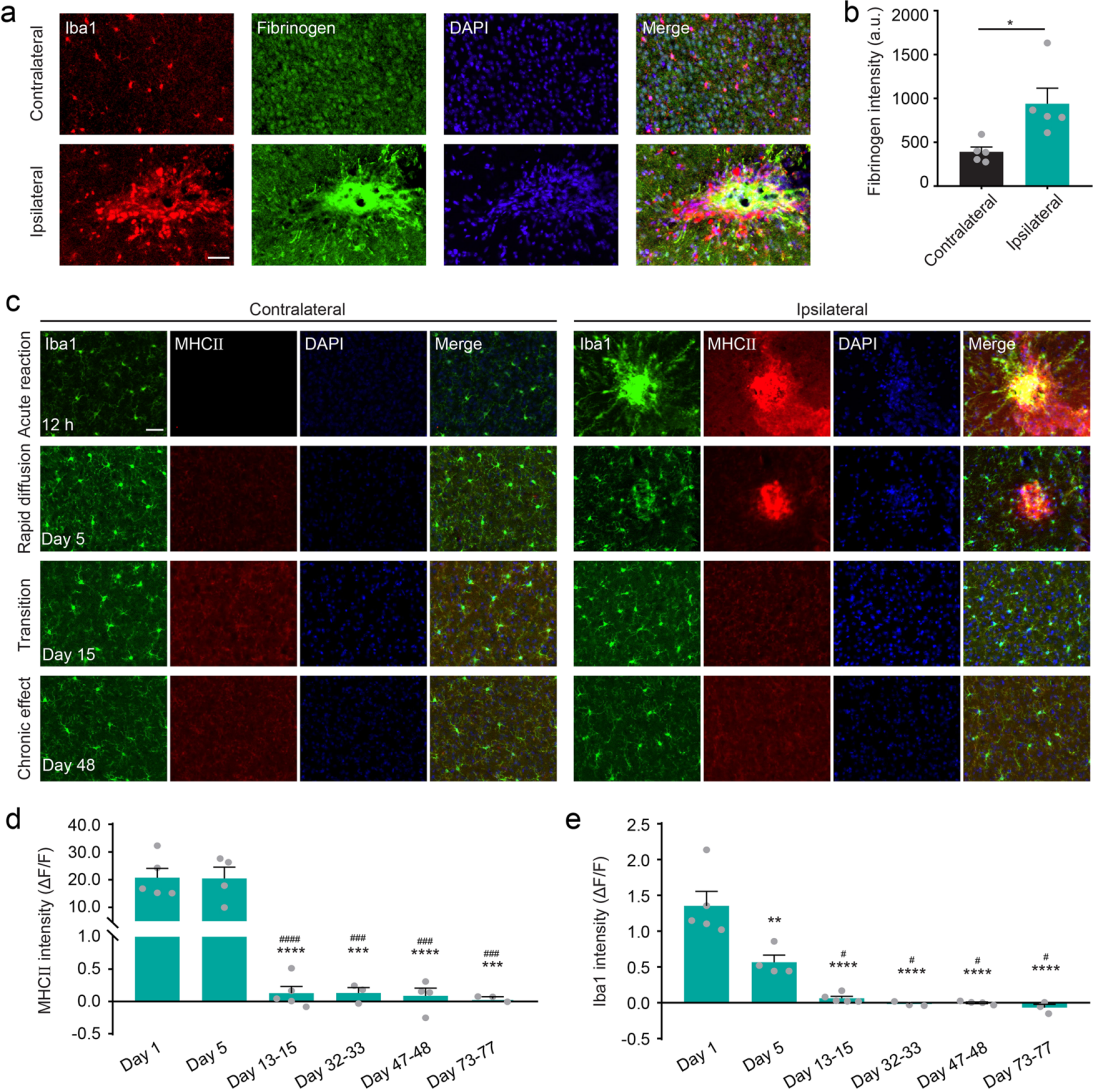
Fibrinogen-induced microglial cluster promotes MHCII expression after vessel occlusion. **a** Representative image of immunostaining for Iba1 (red), fibrinogen (green), and DAPI (blue) in the contralateral and ipsilateral cortex of vessel occlusion mouse in the acute reaction phase. Scale bar, 40 μm. **b** Quantitative analysis of fibrinogen immunostaining in the acute reaction phase (*n* = 5 mice, *P < 0.05, paired *t* test). Error bars, mean and SEM. **c** Representative images of immunostaining for Iba1 (green), MHCII (red), and DAPI (blue) in the contralateral and ipsilateral cortex in the acute reaction, rapid diffusion, transition and chronic effect phase. Scale bar, 40μm. **d**–**e** Quantitative analysis of MHCII expression (**d**) and Iba1 expression (**e**) in the vessel occlusion mice over time (*n* = 3–5 mice, **P < 0.01, ***P < 0.001, ****P < 0.0001 versus day 1; # *P* < 0.05, ### *P* < 0.001, ### *P* < 0.0001 versus day 5, ns indicates no significant difference, one-way ANOVA followed by Tukey’s multiple comparison). Error bars, mean and SEM.

In control experiment, we performed long-term imaging in no laser-induced occlusion mice (Supplementary Fig. 6a). The results show that no microglial cluster occurs and microglia remain in a symmetrical ramified state (Supplementary Fig. 6b; Supplementary Data 2). Therefore, we speculated peri-occlusion microglial cluster in the acute reaction phase may be caused by certain vascular-derived cytokine. It has been reported that stroke is correlated with blood-brain barrier (BBB) injury, which leads to the local extravasation of plasma fibrinogen^38,43–45^. Therefore, we performed immunohistochemistry for fibrinogen. The results show that the presence of fibrinogen deposits in microglial cluster (Fig. 3a), and the intensity of fibrinogen in the ipsilateral is significantly higher than that in the contralateral (Fig. 3b, *P* = 0.02). Therefore, fibrinogen is one of the mechanisms that cause microglial cluster in our study.

The morphology and function of microglia are closely related. Microglial cluster began to diffuse after the acute reaction, and display different morphology characteristics. To explored the potential molecular-level mechanistic changes, we performed immunohistochemistry for major histocompatibility complex (MHCII) (Fig. 3c), an immunoreactivity marker active during proinflammatory signaling response^46^. Immunohistochemical staining confirmed the presence of MHCII, and MHCII colocalized with microglia cluster (Fig. 3c). Quantification analysis shows that the expression of MHCII in the acute reaction and rapid diffusion are comparable (Fig. 3d), although microglial cluster in the rapid diffusion decreased significantly compared with that in the acute reaction (Fig. 3e; Supplementary Data 1). The expression of both MHCII and Ionized calcium-binding adapter molecule 1 (Iba1) in the transition and chronic phase dramatically decreased, and there is no difference during these phases. Together, these results demonstrate that there exists a significant inflammatory response in the early stage of vascular occlusion, and the inflammatory markers greatly decreased in the later stage accompanied with microglial cluster resolving.

### Microglia dynamics in the acute reaction phase

**Fig. 4:**
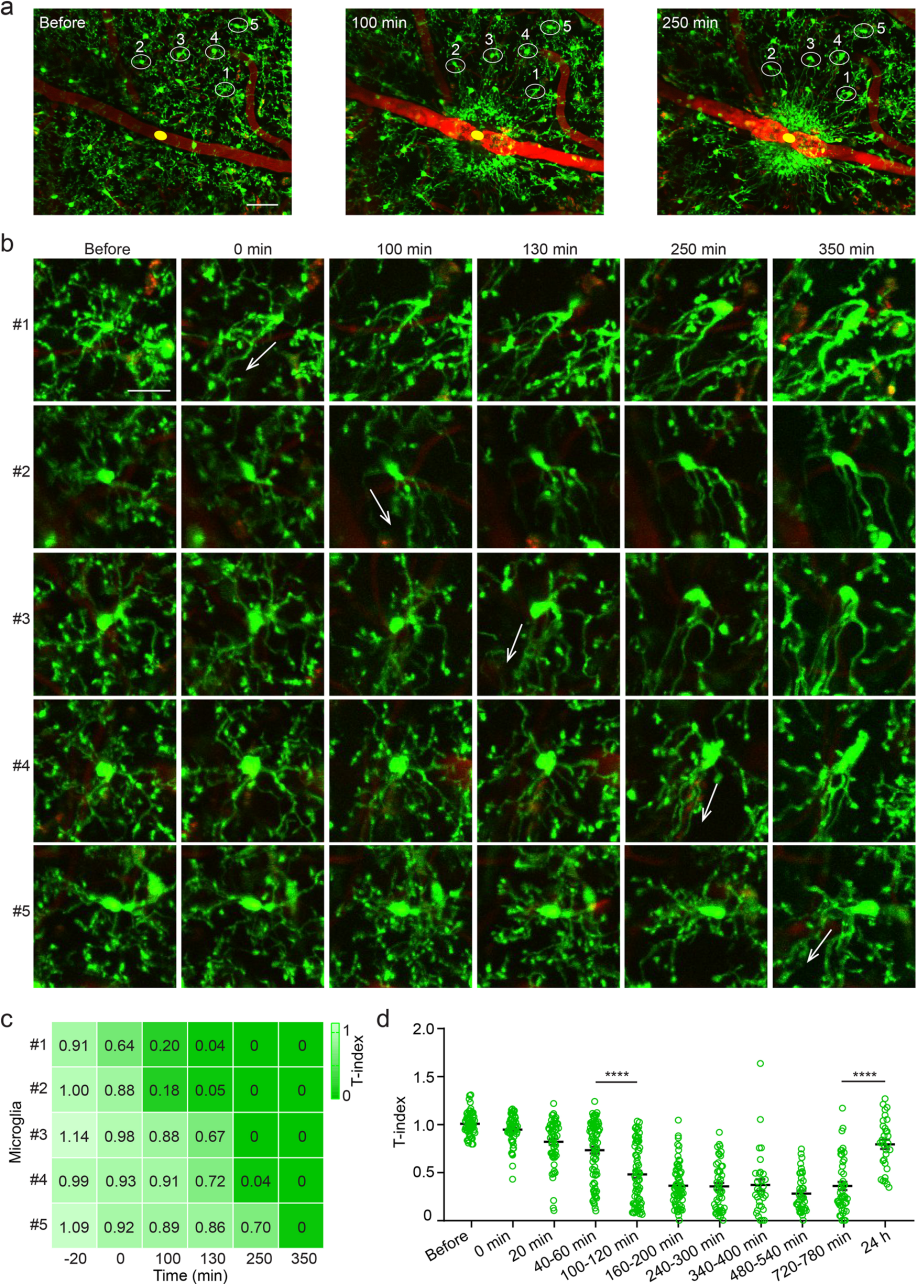
Microglia morphology dynamics in the acute reaction phase. **a** Images showing microglial response to vessel occlusion. Yellow circle indicates occlusion site. White circles indicate five representative microglial dynamics. Scale bar, 50 μm. **b** Microglia processes facing occlusion site elongate, non-occlusion facing process retract. Images show morphology changes of five microglia from near to far from the occlusion site at different time points. Their location was as shown in **a**. Arrows indicate the direction and the time at which microglia processes facing the vessel occlusion site elongate. Scale bar, 20 μm. **c** T-index heatmap of five microglia shown in **b**. The values of T-index at different time points are displayed. **d** Quantification of T-index during 24 h in response to occlusion (*n* = 4 mice, ****P < 0.0001, one-way ANOVA followed by Tukey’s multiple comparison). Error bars, mean and SEM.

Next, we attempted to determine in detail the morphological change characteristics and underlying mechanisms in each phase. Microglia outgrew processes immediately after vessel occlusion, which became obvious 100 min later (Fig. 4a). A hollow spherical containment, which was reported as a protective barrier preventing damage from spreading around^37^, had been formed around the occlusion site. This hollow appearance was also consistent with what was shown in the MCAO model, since few microglia present inside the ischemic core^47^. The processes of the nearby microglia reached the clotted site, forming a solid spherical containment within 250 min (Fig. 4a). During this phase, we found microglia activation based on the distance to the clotted site, from near to far, which took place sequentially. Microglia 1 was the nearest to the occlusion site, microglia 5 the farthest (Fig. 4b). We employed T-index to quantitatively analyze the degree of microglia activation. Before the occlusion, all the five microglia had the appearance of normal morphology with ramified processes. When the blood vessel was blocked, the nearest microglia 1 elongated its occlusion facing process and its non-occlusion facing process retracted at the same time. But the other four microglia retained their ramified processes. In the next few hours, microglia 2 gone through this transition 100 min post occlusion, 130 min for microglia 3, 250 min for microglia 4, 350 min for microglia 5, respectively (Fig. 4b, c). T-index significantly correlated with time after occlusion (Supplementary Fig. 7a), which demonstrates the activation degree of microglia increasing over time.

Compared with T-index at 720–780 min, T-index at 24 h increased significantly (Fig. 4d, *P* < 0.0001), which suggests that the process elongation state has ended 24 h after occlusion. At the same time, the core of solid spherical containment got the most microglia aggregation, while the microglia aggregation in the area far away from the core is greatly reduced (Supplementary Fig. 7b, relative intensity: core = 415.89 ± 68.70, adjacent = 132.04 ± 21.93, near = 25.95 ± 2.90).

**Fig. 5:**
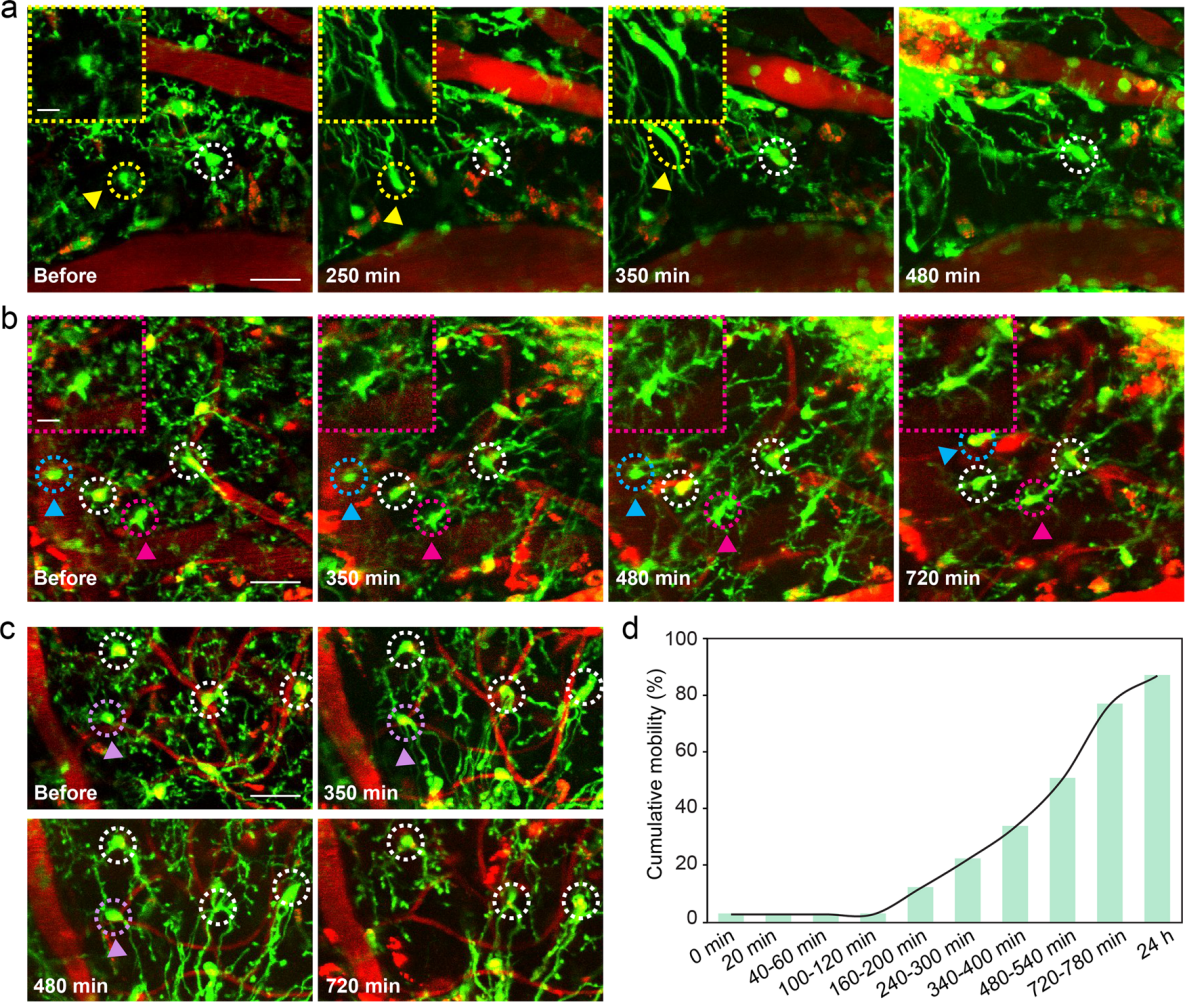
Microglial cumulative motility in the acute reaction phase. **a** Microglial cell body changes from round shape to filamentous-like shape and follows the filamentary path to get into occlusion core. Yellow arrowheads and dashed circles indicate representative microglia that underwent filamentous change. Insets are magnified images. White dashed circle indicates stationary microglia. Scale bars, 30 μm (overview) and 10 μm (inset). **b** Cyan and rosy arrowheads and dashed circles showing microglia with no filamentous deformation of the cell body migrating toward occlusion site. White dashed circle indicates stationary microglia. Scale bars, 30 μm (overview) and 10 μm (inset). **c** Purple arrowheads and dashed circles indicate disappeared microglia with no migration occurrence. White dashed circle indicates stationary microglia. Scale bar, 30 μm. **d** Cumulative mobility of microglia in response to occlusion during 24 h. Data are plotted as mean value, *n* = 4 mice.

Although microglial processes responded very quickly, microglial cell bodies motility response was much slower. We found cell bodies migration was performed in two distinct ways (Fig. 5a–c), including the cell bodies deformation migration and cell bodies nondeformation migration. Cell bodies deformation migration was named filamentous-like movement (Fig. 5a). In this way, the cell body was stretched into filaments and then migrated into the occlusion core. Cell bodies non-deformation migration were divided into two categories. The first category underwent obvious displacement (Fig. 5b), and cell body in another category vanished while adjacent cell bodies kept stationary (Fig. 5c). The cumulative motility was only 2.52% ± 1.51 at 100–120 min after occlusion (Fig. 5d), which suggests the majority of cell bodies keep stationary during this phase. However, the cumulative motility rate increased rapidly reaching 76.71% ± 3.89% at 720–780 min. Cumulative motility rate was 86.87% ± 3.89% at 24 h and suggests that almost all microglia have been translocated. In previous reports, few studies have mentioned the movement of the microglia cell body. Here, our results provide an accurate and concise clarification about it.

### Microglia dynamics in the rapid diffusion phase

**Fig. 6:**
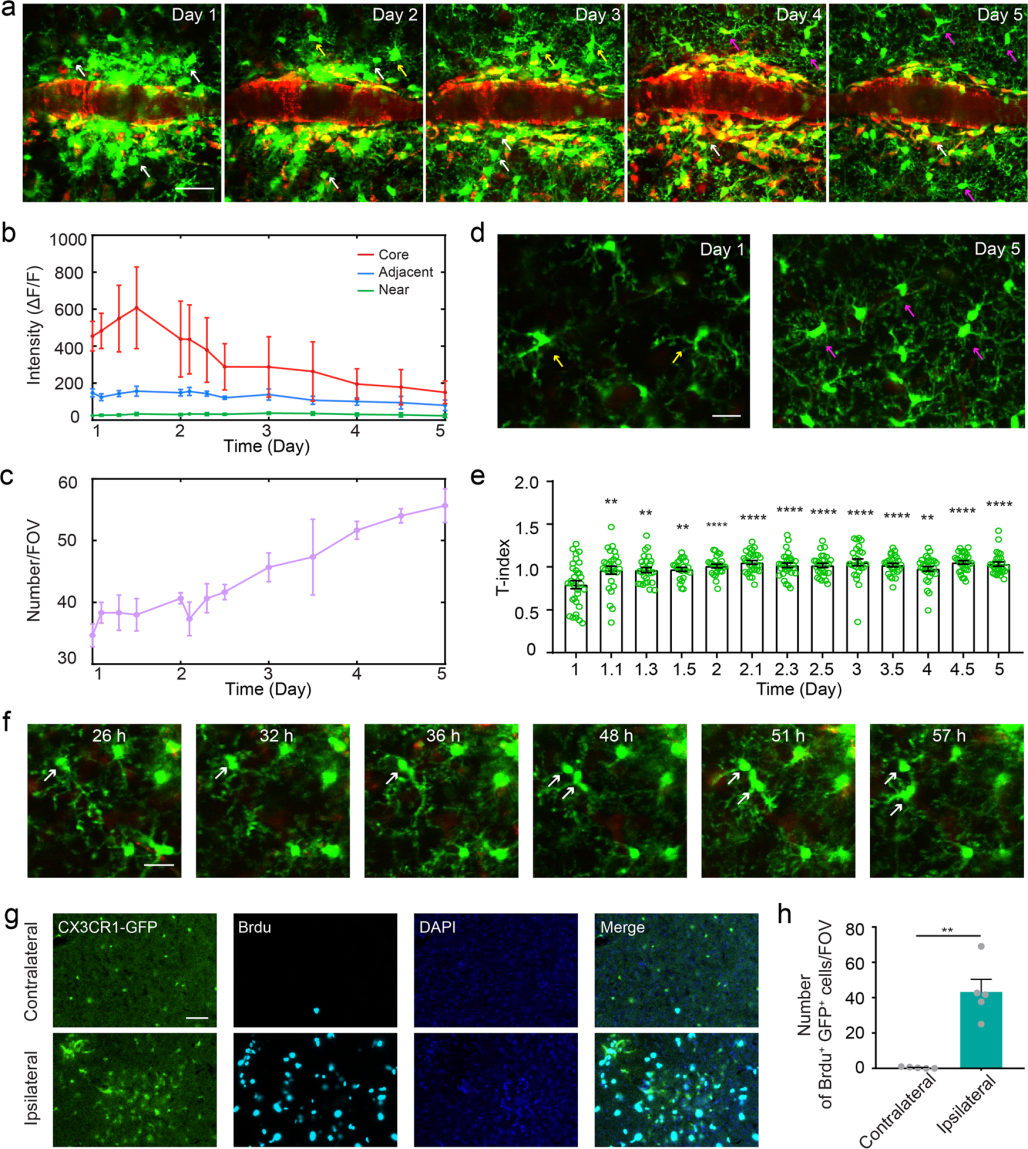
Microglia dynamics in the rapid diffusion phase. **a** Repetitive in vivo imaging in the same FOV after occlusion shows a resolving microglia cluster over time. White arrows indicate microglia of round shape with few processes. Yellow arrows indicate microglia with asymmetric processes. Purple arrows indicate microglia with well-proportioned ramified process. Scale bar, 40 μm. **b** Persistent relative intensity variance at different time points from the inner to the outer area as shown in Fig. 2b (*n* = 3 mice). Data are plotted as mean ± SEM. **c** The number variance of microglia from day 1 to day 5 after occlusion (*n* = 3 mice). Data are plotted as mean ± SEM. **d** Images showing difference in the number and morphology of microglia at day 1 and day 5 after occlusion. Compared with that at day 1 (yellow arrows), the symmetry of microglia process is significantly improved at day 5 (purple arrows). Scale bar, 20 μm. **e** Quantification of T-index at different time points (*n* = 3 mice, ** *P* < 0.01, ****P < 0.0001 versus T-index on day 1, one-way ANOVA followed by Tukey’s multiple comparison). Error bars, mean and SEM. **f** Images showing proliferative microglia during repeated imaging in vivo. Scale bar, 20 μm. **g** Representative images of immunostaining for Brdu (cyan) and DAPI (blue) in the contralateral and ipsilateral cortex of vessel occlusion CX3CR1-GFP mouse in the rapid diffusion phase. Scale bar, 40 μm. **h** Quantitative analysis of Brdu^+^GFP^+^ cells in the contralateral and ipsilateral cortex as shown in **g** (*n* = 5 mice, **P < 0.01, paired *t* test). Error bars, mean and SEM.

After having known the characteristics of the acute response of microglia to vessel occlusion, we repeatedly imaged to investigate what happens to the microglia after cluster formation. At this phase, we can see massed outlines of microglial cell bodies with few short processes around the occlusion site at day 1 (Fig. 6a). Then these cell bodies continued to move out of the core, and the process began to be abundant until the majority of them finally became the ones with normal symmetrical process at day 5.

Due to the removal of the cell bodies from the core, the relative fluorescence intensity of the occlusion core decreased from 608.23 ± 220.98 at day 1.5 to 150.42 ± 62.00 at day 5 (Fig. 6b). Unexpectedly, no obvious fluctuation was observed in the intensity of the outer area from the occlusion core, which means that the emerged microglia migrated continuously to the remote area to maintain a balance of spatial distribution. The total number of microglia in the FOV also gradually increased, from 34.67 ± 1.86 at day 1 to 55.67 ± 2.73 at day 5 (Fig. 6c). In terms of morphology, we found that the microglia were still activated at day1 (Fig. 6d) with T-index 0.79 ± 0.05, followed by a significant increase in the T-index at day 1.1 (Fig. 6e, 0.96 ± 0.05, *P* < 0.01). No significant difference in T-index was observed from day 1.1 to day 5, which indicates the morphology of microglia has been stable.

We found that microglial proliferation contributed to the increased microglial number. Our in vivo imaging described in detail microglial proliferation procedure (Fig. 6f). In addition, we also performed immunohistochemistry for bromodeoxyuridine (Brdu) (Fig. 6g), a marker commonly used in the detection of proliferating cells. Quantitative analysis demonstrates the number of Brdu^+^GFP^+^ cells in the ipsilateral peri-occlusion area is significantly higher than that of contralateral (Fig. 6h, contralateral vs ipsilateral: 0.4 ± 0.17 vs 43.2 ± 6.42, *P* = 0.0042). At the same time, microglia disappeared in their original position and also appeared in the original blank position (Supplementary Fig. 8a, b). Through these various morphological changes discussed above, the peri-occlusion microenvironment was renewed.

### Microglia dynamics in the transition phase

**Fig. 7:**
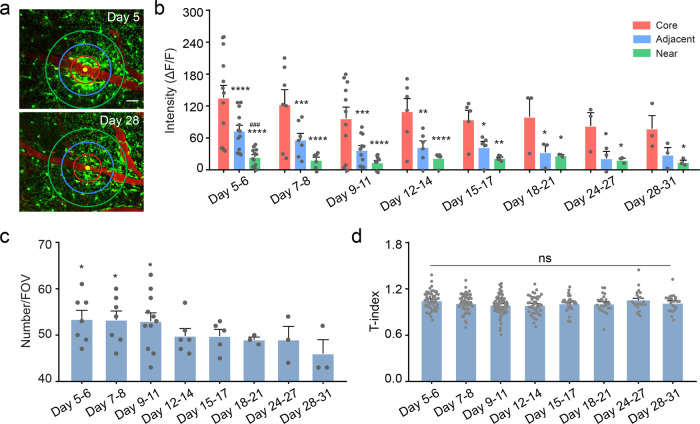
Microglia dynamics in the transition phase. **a** Representative image in the transition phase. Scale bar, 40 μm. **b** Quantification of relative fluorescence intensity over time from the inner to the outer area as shown in Fig. 2b (*n* = 3 mice, ### *P* < 0.001 versus the adjacent area; *P < 0.05, **P < 0.01, ***P < 0.001, ****P < 0.0001 versus the core area, one-way ANOVA followed by Fisher’s LSD multiple comparison). **c** Time-course analysis of microglia number in the transition phase (*n* = 3 mice, *P < 0.05 versus day 28–31, one-way ANOVA followed by Fisher’s LSD multiple comparison). **d** Quantification of T-index in the transition phase (*n* = 3–4 mice, ns indicates no significant difference, one-way ANOVA followed by Tukey’s multiple comparison). Data are presented as mean and SEM.

Next, we studied the microglia dynamics in the later phases of vascular occlusion. During the transition phase, although accumulation of perivascular microglia decreased (Fig. 7a), microglia density was still slightly higher in proximity to occlusion core. Therefore, the relative fluorescence intensity of occlusion core was significantly higher than that of the outer area (Fig. 7b). In terms of microglia number, it decreased significantly at day 28–31 (46.00 ± 3.00) compared with that at day 5–6 (53.43 ± 1.93) (Fig. 7c). There was no difference in the morphology of individual microglia (Fig. 7d). These results show that the pathological effects caused by vascular occlusion still exist in the transition phase.

### Microglia recover to resting state in the chronic effect phase

**Fig. 8:**
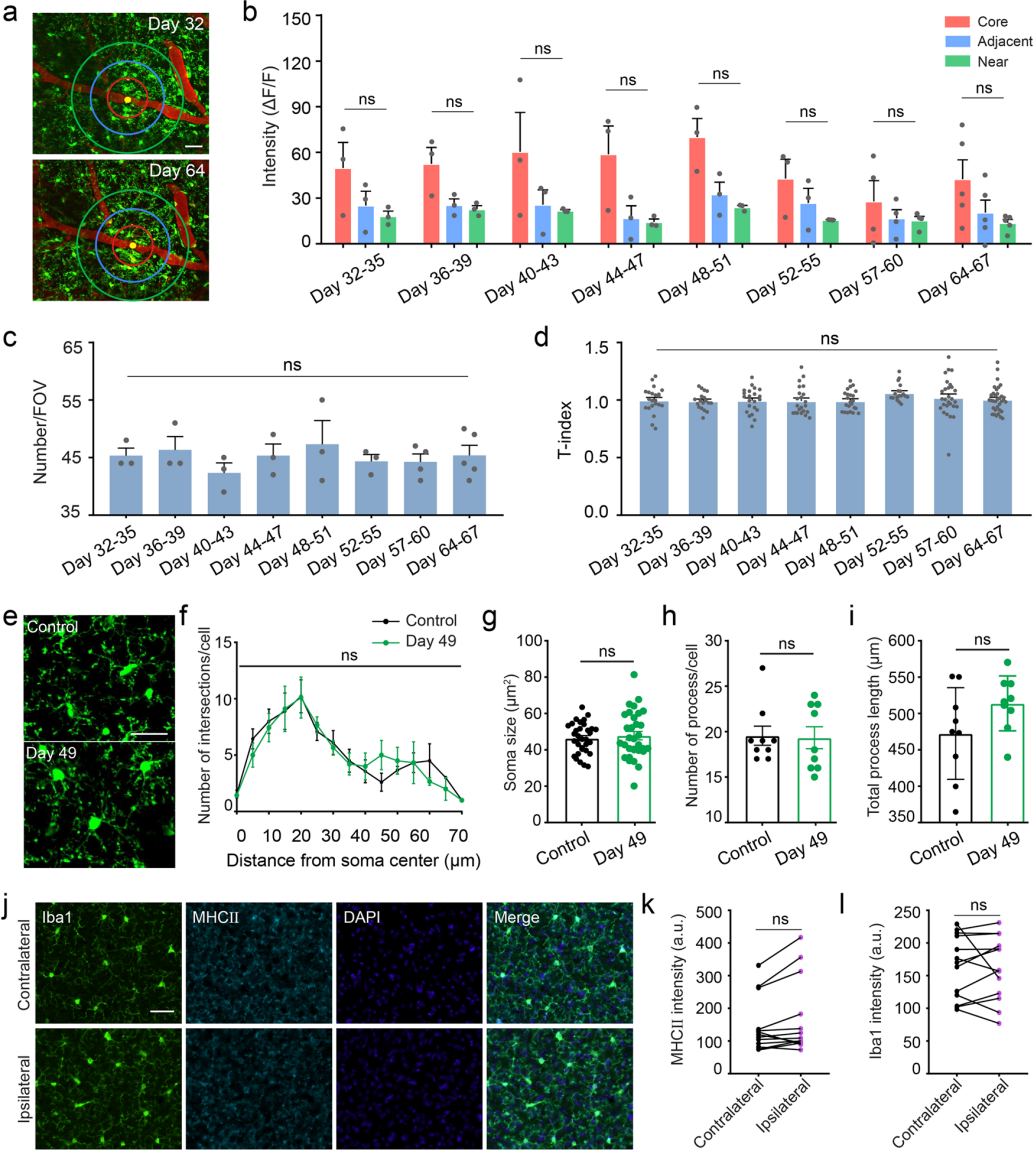
Microglia recover to resting state in the chronic effect phase. **a** Representative in vivo image in the chronic phase. Scale bar, 40 μm. **b** Quantification of relative fluorescence intensity in the chronic phase from the inner to the outer area as shown in Fig. 2b (*n* = 3 mice, one-way ANOVA followed by Fisher’s LSD multiple comparison). **c** Time-course analysis of microglia number in the chronic phase (*n* = 3 mice, one-way ANOVA followed by Fisher’s LSD multiple comparison). **d** Quantification of T-index in the chronic phase (*n* = 3 mice, one-way ANOVA followed by Tukey’s multiple comparison). **e** Representative microglial morphology images in vivo of control and vessel occlusion mice. Scale bar, 30 μm. **f** Sholl analysis of microglial morphology in vivo in control and vessel occlusion mice (*n* = 3 mice, Two-way ANOVA). **g**–**i** Quantification of cell morphometry, including soma size (**g**), total number of process (**h**) and process length (**i**) of microglia (*n* = 3 mice, unpaired *t* test). **j** Representative images of immunostaining for Iba1 (green), MHCII (cyan) and DAPI (blue) in the contralateral and ipsilateral cortex of vessel occlusion mouse in the chronic effect phase. Scale bar, 40 μm. **k**–**l** Quantitative analysis of MHCII (**k**) and Iba1 (**l**) immunostaining intensity as shown in **j**, *n* = 4 mice, paired *t* test. Data are presented as mean and SEM, ns indicates no significant difference.

In the chronic phase, microglial cluster resolved (Fig. 8a). There was no significant difference of relative fluorescence intensity between inner area and outer area (Fig. 8b). Microglial cell number and morphology T-index within the FOV also showed no obvious fluctuations (Fig. 8c, d).

To further determine whether microglia recover to their original physiological state prior to occlusion, we analyzed the morphology of individual in vivo microglia in detail (Fig. 8e). The results show that there is no significant difference between control and occlusion mice in number of intersections (Fig. 8f), soma size (Fig. 8g, control vs day 49: 44.85 ± 1.47 vs 47.84 ± 2.24, *P* = 0.62), number of process (Fig. 8h, control vs day 49: 19.56 ± 1.00 vs 19.33 ± 1.13, *P* = 0.89) and total process length (Fig. 8i, control vs day 49: 473.50 ± 19.78 vs 513.93 ± 11.87, *P* = 0.11). We also performed immunohistochemistry for MHCII and Iba1 (Fig. 8j). The expression of MHCII and Iba1 are comparable in the contralateral and ipsilateral cortex (Fig. 8k, l). Besides, we re-occluded the same vessel (Supplementary Fig. 9a-c). The diffused microglia re-cluster to the occlusion site again (Supplementary Fig. 9d). Together, these results indicate microglia recover to resting state both in the morphology and function in the chronic effect phase.

## Discussion

In this paper, we explore the in vivo spatiotemporal dynamics of microglia during single vessel occlusion and recanalization up to 73 days. We find that there are four phases of distinct microglia dynamics characteristics, including acute reaction, rapid diffusion, transition and chronic effect. In the acute reaction phase (0–24 h), microglia are rapidly activated manifested by processes elongation towards occluded sites as expected. Furthermore, we identified the morphological changes and diverse displacement of cell bodies that occur much later compared to that of processes. In the rapid diffusion phase (day 1-day 5), proliferative microglia move out of the cluster, which leads to decreasing in fluorescence intensity of occlusion core and increasing in microglial number. Immunohistochemical staining demonstrates fibrinogen-induced microglial cluster promotes MHCII expression in the acute reaction and rapid diffusion phase. In the transition phase (day 5-day 31), microglia cluster still exist, since the fluorescence intensity of the occlusion core is significantly higher than that of distant area. Finally, microglia recover to resting state both in the morphology and function in the chronic phase (>=day 32). Compared with previous research focused on the acute reaction phase, our study well investigated the microglial complicated morphological transformation characteristics and potential mechanisms in the long-term scale.

It is worth noting that microglia processes are the main participants moving toward to injury site in the acute reaction phase. By tracking the same microglia, we demonstrate that the motility of microglial cell bodies did not start to increase positively until 120 min after the occlusion. Since imaging sessions also has a certain time interval, we found that a small part of the cell bodies, either close to the occlusion core or becoming filamentous ones, could migrate into the occlusion core. Rapid increasing in cumulative mobility is primary contributed by the disappearance of the cell body, so it is ambiguous that whether the cell bodies have migrated to the occlusion site. Strikingly, in the rapid diffusion phase, we found plenty of proliferative microglia with clear round shape cell body move out from the cluster and redistribute their territory to regulate brain parenchyma microenvironment. Therefore, we confirm microglial proliferation occurs during the phase of interaction between microglia cluster and occluded vessel. Together, these results suggest there are three important concerns in the future study. First, more in vivo imaging should be performed especially in the acute reaction and rapid diffusion phase to know more about microglia dynamics, including microglial soma and process. Secondly, more focus should be on these proliferative microglia. In a previous genetic cell ablation study, microglial micro-clusters prove to be highly proliferative and single cells migrate away from the cluster once steady state is achieved^35^. Interleukin-1 receptor (IL-1R) on microglia participates in this cluster-style proliferation process. Besides, GW2580 administration, a colony-stimulating factor-1 receptor inhibitor that inhibits microglia proliferation after spinal cord injury, improves motor function recovery in mice and nonhuman primates^48^. If IL-1 signaling pathway can be manipulated or GW2580-treatment is performed in our future study, it will be very interesting and important for us to explore the role of these proliferation microglia. Thirdly, some of the proliferative cells are not microglia in our study. Further confirmation of the cell type of these proliferative cells is necessary.

It has been reported in acute experimental research that ATP mediates a rapid microglial chemotaxis requires P2Y receptor through either its associated potassium channels activation or extracellular nucleotides^49,50^. However, the chemotaxis mechanism of microglia is not the same in different models of neurological diseases, and the detrimental or protective role of microglia remains highly controversial in neurological diseases^34,47,51,52^. In autoimmune encephalomyelitis mice, rapid microglial responses toward the vasculature lead to axonal damage^53,54^. Whereas, under the pathological BBB breakdown condition, perivascular microglial processes play a protective role in the maintenance of BBB integrity following cerebrovascular damage^55^. In our study, immunohistochemistry results show that MHCII, a M1-assosicted microglial activation marker, colocalize with microglial cluster in the acute reaction and rapid diffusion phase. Substantial studies have shown that upregulated MHCII is always accompanied with the release of inflammatory factors^56,57^, such as tumor necrosis factor-α, interleukin-1β, and interferon-γ; while the expression of anti-inflammatory markers, such as mannose receptor (CD206) and arginase 1, remain at baseline level. This suggests microglia may play a detrimental role in the early stage. In the transition phase and chronic effect phase, there is no difference in the MHCII expression between the contralateral and ipsilateral cortex, which suggests related pro-inflammatory cytokines return to baseline level alleviating the inflammatory environment. Boosting T lymphocytes can shift microglia toward the M2 phenotype and ameliorate intracerebral hemorrhage-induced inflammatory injury^17^, which may be one potential method for us to manipulate microglia toward M2 phenotype in the early stage of vessel occlusion. In the end, it is particularly worth noting that microglia experience an early neuroprotective M2 phenotype, followed by a transition to a proinflammatory M1 phenotype in peri-infarct regions^58,59^. Microglia responding to ischemic injury dynamically suggests corresponding treatments for different pathological phases should be considered instead of simply suppressing microglia. Recanalization vessel have been malformed although microglia recover to the resting state in our present study. We speculate there exists factors involved in the long-term pathological progression other than microglia. These all suggest us more molecular-level exploration should be performed, especially in the transition and chronic effect phase in the future study.

## Methods

### Transgenic mice

Heterozygous CX3CR1^GFP/+^ mice and WT C57BL/6 J mice were used in our experiments. In CX3CR1^GFP/+^ mice, microglia are labeled with an enhanced green fluorescent protein (EGFP), which provides high contrast in microglia soma and processes imaging. The animals were housed in a temperature-controlled and humidity-controlled room with a 12 h/12 h light/dark cycle. Mice were allowed free access to purified water. The appropriate guidelines for the care and use of laboratory animals were approved by the Committee for Animal Experiments of Zhejiang University.

### Implantation of chronic cranial window

Cranial window implantation above the mouse cortex was for in vivo laser ablation and imaging. Transgenic CX3CR1^GFP/+^ mice (8 to 10 weeks old) were anesthetized with isoflurane (1.5%–2.5%) and placed in a stereotactic frame with a heating pad (36–37.5 °C). Depth of anesthesia was assessed by monitoring pinch withdrawal and respiration rate. Eyes were protected with ointment.

The scalp was incised with scissors. Lidocaine (2%) was administrated locally. Then we carefully removed the muscle and connective tissue above skull with a disposable blade or needle. A small craniotomy (approximately 3 mm × 3 mm) was performed using a high-speed drill with a small-tip steel burr (0.5 mm in diameter), centered at 1.7 mm lateral to the midline and 2 mm posterior to bregma. A double-layered coverslip consisted of a small coverslip (3 mm in diameter) attached to a large one (6 mm in diameter), which was embedded and sealed with dental cement. The small layer fitted snugly into the craniotomy, and the large one was attached to the polished skull.

The last step used dental cement to fix the head plate on the skull. The mice were placed on a heating pad until they were fully awake. Antibiotics injections were administered after surgery for at least one week. Mice were housed for about 4-weeks recovery from the surgery.

### In vivo long-term imaging with two-photon microscope

EGFP-labeled microglia were imaged by two-photon microscope through a small craniotomy as described above. By raster scanning a femtosecond-pulsed laser beam (Chameleon Ultra II, Coherent) via standard galvanometer raster scanning with a moving in vivo microscope (Bruker Corporation), two-photon imaging was performed. A 16×/0.8-NA water-immersion objective (Nikon) was used in all the experiments. The Ti-sapphire laser was set at the excitation wavelength of 920 nm for both EGFP-expressing microglia and Texas red-labeling blood vessels imaging. A stack of image planes (1064 × 1064 pixels) with a step size of 2 μm was acquired using the water-immersion objective at a zoom of 2.0. The maximum imaging depth was ~350 μm from the pial surface. Images were acquired with low laser power (<50 mW at the sample).

To visualize the vasculature and the motion of red blood cells (RBCs) with TPLSM, we injected 7 mg/kg Texas Red dextran (70,000 MW, neutral; Thermo) in 0.9% NaCl intravenously. Line scans were used along single vessels with a maximum scan rate of 5 kHz to quantify RBCs velocity. RBCs movement resulted in dark diagonal streaks in the image with a slope that was inversely proportional to the RBCs velocity.

### Vascular occlusion with two-photon laser

The excitation source for the photostimulation path was a femtosecond-pulsed laser fixed at 1070 nm (total output, 2 watts; pulse width, 55 fs; Coherent). We focused a two-photon laser beam (~15 μm in diameter) on the vessel through the cranial window to create a highly localized injury, and the laser power was set at 80–200 mW. The photostimulation beam was paused at the anticipated position of the vessel for approximately 1–30 s to create a small injury site, which was indicated by a bright fluorescent circle around the focal point of the beam. We assessed blood flow and vascular appearance from the real-time TPLSM images to adjust the laser power, and pulse duration until the final establishment of vascular occlusion.

### Brdu admistration

For the in vivo proliferation study, bromodeoxyuridine (Brdu; Sigma, B5002) was dissolved in 0.9% saline at a concentration of 10 mg/mL. The mice received Brdu (100 mg/kg) injections daily from day 0 to day 3 after occlusion, and were sacrificed 2 h after the final injection.

### Immunohistochemistry and image analysis

Mice were deeply anesthetized with isoflurane (~2 min) and then perfused transcardially quickly with ~25 mL of ice-cold 1× PBS and ~ 20 mL 4% paraformaldehyde solution. Brains were removed and post-fixed in 4% paraformaldehyde solution at 4 °C overnight, and then immersed in 30% sucrose solution at 4°C for 2‒3 days for dehydration. Brains were then frozen and cut into 30 μm horizontal slices with a freezing microtome system (CryoStar NX50, Thermo) at −20°C. Brain slices were immersed in antifreeze solution and stored at −20°C. For staining, the brain slices were washed 4× with PBS for 5 min and then were incubated in a blocking solution consisting of 10% normal donkey serum and 1% Triton X-100 diluted in 1× PBS at room temperature for 2 h. Slices were then incubated in primary antibodies overnight at 4°C with 1% Triton X-100 and 10% donkey serum. The primary antibodies used were as follows: rabbit anti-fibrinogen (1:200, Abcam, ab34269), mouse anti-MHCII (1:200, Abcam, ab23990), rabbit anti-Iba1 (1:1000, Wako, 019-19741), goat anti-Iba1 (1:500, Wako, 011-27991), rat anti-Brdu (1:200, Abcam, ab6326). Next, slices were washed 4 × with PBS for 5 min and incubated with secondary antibodies for 2 h at room temperature in a dark place. The second antibodies used were as follows: donkey anti-rat 647 (1:500, Abcam, ab150155), donkey anti-rabbit 488 (1:500, Abcam, ab150073), donkey anti-goat 555 (1:500, Abcam, ab150134), donkey anti-mouse 647 (1:500, Abcam, ab150111). Finally, all brain slices were mounted with antifade medium containing DAPI (Beyotime) to label the nuclei.

For Brdu staining, brain slices were washed 4 × with PBS, incubated with 2 M HCL for 30 min at 37 °C, rinsed with 0.1 M borate buffer (PH 8.5) at room temperature for 30 min, washed 6 × with PBS, and then subjected to above staining procedure.

For quantification of immunohistochemistry, slices images were taken using a VS120 Virtual Slide Microscope (Olympus). The fluorescence intensity of MHCII and Iba1 at different time points was given by *ΔF/F* = *(F(ipsilateral) – F(contralateral))/ F(contralateral)*.

### Two-photon imaging data analysis

Images were processed by ImageJ software. All z-stacks of images were projected along the z-axis to produce two-dimensional maximum intensity projection images within the imaged volumes. Function as a morphological parameter, the microglial territory areas were quantified by circumscribing the outline of the ends of microglia processes with a smooth polygon tool in ImageJ. Interactions of Sholl analysis, soma size, number of process and process length were also quantified with ImageJ.

To account for signal intensity differences that arose from different long-term imaging experiments, the microglia fluorescence image was separated from the two-channel images and then signal intensity measurements were conducted. We measured microglia fluorescence intensity changes through four concentric circles from the inner area to the outer area (core, adjacent, near), and the fourth outermost circle was regarded as baseline fluorescence value *F*_*0*_. We measured the mean gray value in each circle as *F(t)*. The relative fluorescence intensity of all the circular area was therefore given by *ΔF/F* = *(F(t) – F*_*0*_ *(t))/ F*_*0*_ *(t) × 100*.

The blood vessels were loaded with fluorescent dyes that imaging the outline of perfused vessels. RBCs were displayed as dark particles in the bright background. RBCs velocity were measured with MATLAB 2016a (MathWorks, USA). Vessel diameter and morphology analysis were measured manually with ImageJ.

To evaluate the changes in microglia morphology, we quantitatively analyzed the transitional stage morphology index (T-index)^60^. The T-index was calculated based on the length of the longest microglia process facing the injury blood vessel (n) and the length of the longest microglia process facing away from the injured blood vessel (f). The morphology index was therefore given by *T-index* = *(f - n)/ (f* + *n) + 1*.

For cell bodies cumulative motility analysis, we analyzed microglia in the field of view (FOV) within a volume of 410.55 μm × 410.55 μm × 20 μm. Individual microglia was identified and marked with numbers before vessel occlusion. If the cell bodies maintain their position by comparing the surrounding landmark objects (such as blood vessels), they are considered to be static. If they are not present or exist in a position greater than 10 μm based on the previous image, they are considered to be migratory. Cumulative motility was determined as the number of migratory cells divided by the total number of cells before occlusion × 100%.

### Curve fitting of microglial fluorescence intensity changes

S-curve^39,61^ and diffusion-consumption equation^41,42^ were used to fit the fluorescence intensity changes over time. The S-curve equation is:

1 $${F}_{1}({{{{{\rm{t}}}}}})=\frac{A}{1+B{e}^{-\gamma t}}$$

where *F*_*1*_ (t) (fluorescence intensity) is the dependent variable for the S-curve, *t* is the time, *B* and *γ* are model parameters, while *A* is the carrying density of microglia in this study.

Immune response-induced cytokine diffusion and consumption is governed by a diffusion-consumption mechanism^41^. Fluorescence intensity has a power function relationship with time. We thus modified the equation as follows:

2 $${F}_{2}({{{{{\rm{t}}}}}})=\frac{K}{{\left(t+\triangle t\right)}^{2}}+C$$

where *F*_*2*_ (t) (fluorescence intensity) is the dependent variable, *t* is the time, *K*, *Δt* and *C* are model parameters.

The parameter values of the two equations of core, adjacent, near (from the inner area to the outer area based on occlusion core) are shown in Table 1. The fitting curves were smoothly using algorithm processing.

**Table 1:** Parameter values for curves fitting in our experiment.

Area	*A*	*B*	*γ*	*K*	*Δt*	*C*
Core	612.00	23.00	9.28	1950.00	0.47	50.00
Adjacent	180.00	23.13	8.04	2250.00	2.30	25.00
Near	37.75	23.13	5.24	1150.00	4.82	15.00

### Statistical analysis and reproducibility

We used Prism (GraphPad) and Excel (Microsoft) to perform statistical analysis. Statistical test used, test statistics and the P values are shown in corresponding figure legends. Statistical significance was calculated using one-way ANOVA followed by Tukey’s multi-group comparisons or Fisher’s LSD multiple comparisons, two-way ANOVA test, unpaired *t*-test and paired *t*-test. Groups were identified statistically different at *P* < 0.05. When the *P*-value was greater than 0.05, it is considered as non-significant (ns).

### Reporting summary

Further information on research design is available in the Nature Research Reporting Summary linked to this article.

## Supplementary information

Supplementary Information

Description of Additional Supplementary Files

Supplementary Data 1

Supplementary Data 2

Reporting Summary

## Data Availability

All data needed to evaluate the conclusions in the paper are present in the paper and/or the Supplementary Materials. Source data are provided with this paper.

## Code availability

The codes supporting the current study have not been deposited in a public repository, but are available from the corresponding author upon request.
