# Supplementary material for: Long-term microglial phase-specific dynamics during single vessel occlusion and recanalization
Source: Commun Biol. 2022 Aug 19;5:841. doi: 10.1038/s42003-022-03784-0 (PMC9391347; doi:10.1038/s42003-022-03784-0)
Supplement: Supplementary file 1 — Supplementary Information [file 42003_2022_3784_MOESM1_ESM.pdf]

Supplementary Materials

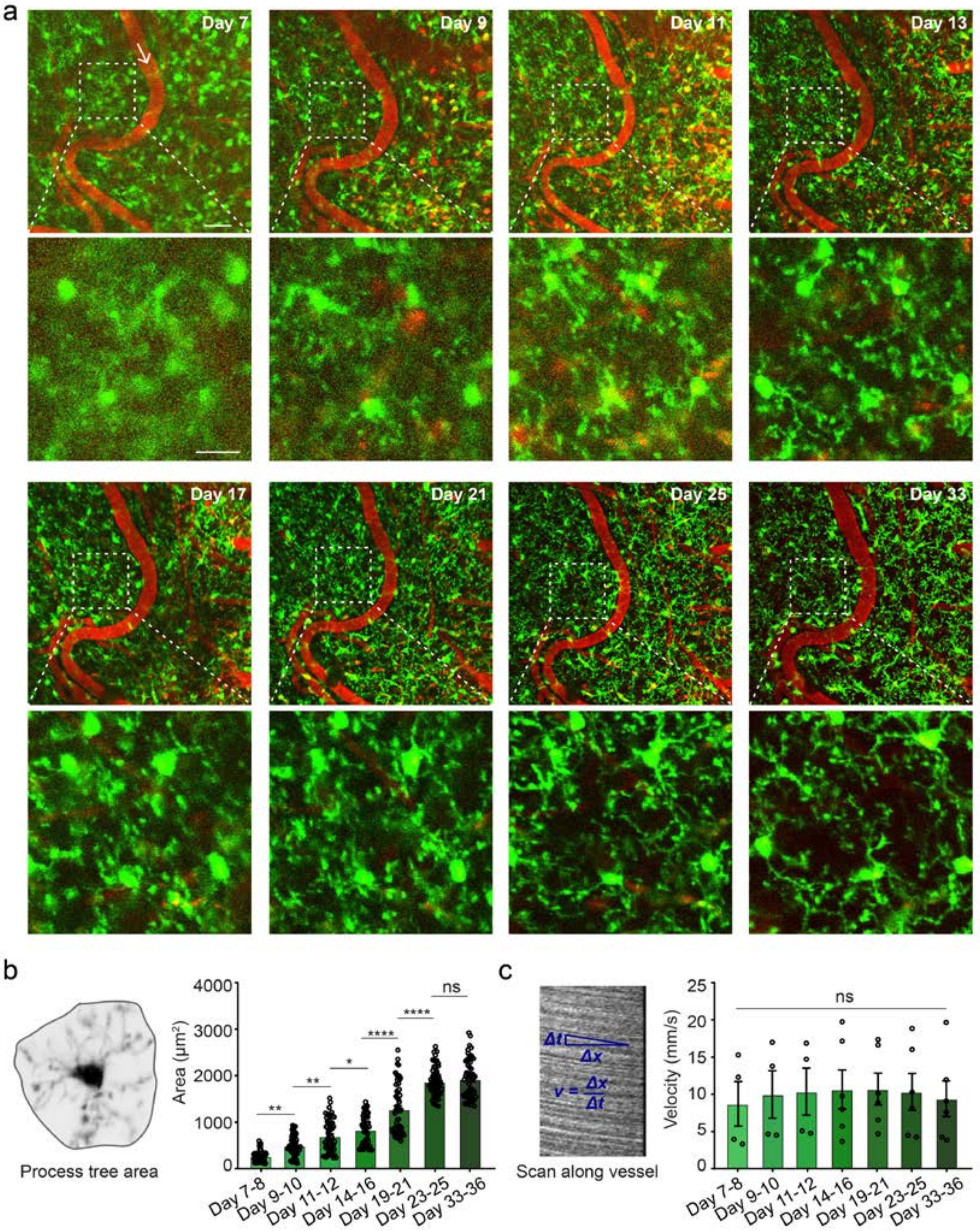

Supplementary Figure 1: Long-term *in vivo* imaging of transgenic CX3CR1-GFP mouse after cranial window implantation.

a Maximum intensity projection of 3D z-stack images showing microglia (green) and blood vessel (red) from day 7 to day 33 after cranial window implantation surgery. White arrow indicates the blood flow direction. The stacks are 20 μm in depth (2 μm stepsize). White square shows higher magnifications of microglia morphology at different time points. Scale bars, top: 50 μm; bottom: 20 μm.

b Process tree area is defined by connecting the outer points of the microglia process arbor and quantification of process tree area on different days. Values are mean and SEM (n = 385 microglia from 4 mice, $***P < 0.001$, ns indicates no significant difference, one-way ANOVA followed by Fisher's LSD multiple comparison).

c Blood flow velocity is calculated from the angle of the streaks and quantification of blood flow velocity on different days. Values are mean and SEM (n = 3 vessels from 3 mice, ns indicates no significant difference, one-way ANOVA followed by Fisher's LSD multiple comparison).

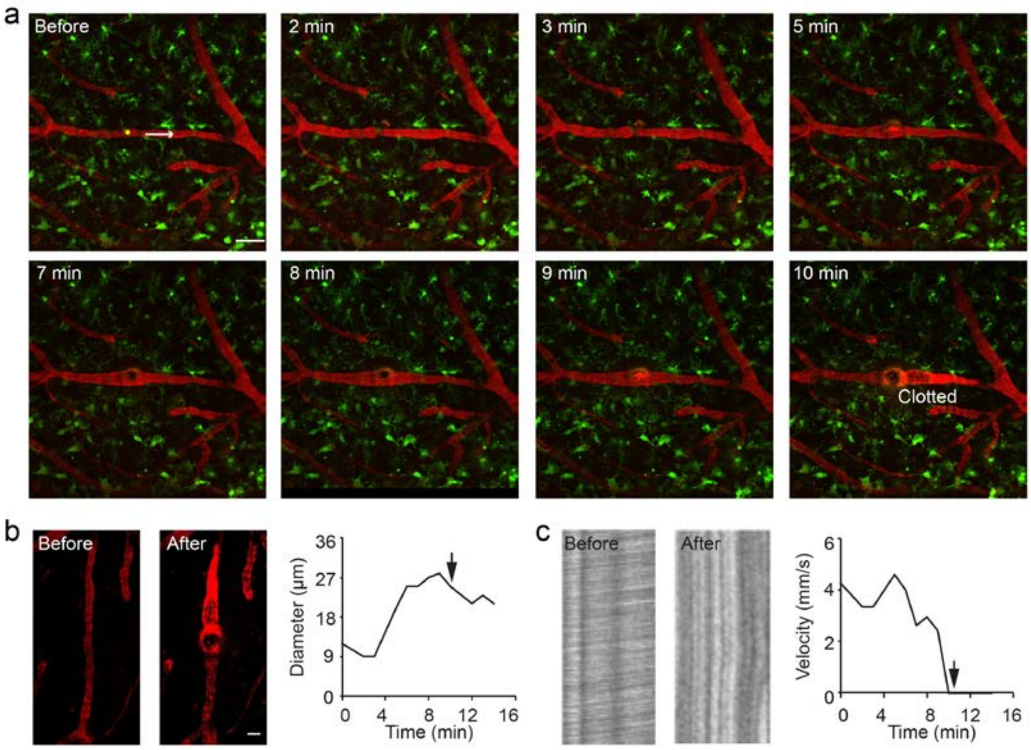

Supplementary Figure 2: Intravascular occlusion in the intact target vessel.

a Temporal sequence of images during intravascular occlusion formation. Yellow circle indicates laser irradiation site. White arrow indicates the blood flow direction. Scale bar, 50 µm.

b Images showing changes in blood vessel diameter before and after occlusion. Vessel diameter graph from representative experiment showing vessel diameter change during occlusion procedure. Arrows indicate vessel occlusion. Scale bar, 20 µm.

c Stack of line scans from the vessel shown in **b** before and after occlusion with moving RBCs represented by dark bands. Vessel velocity graph from representative experiment showing vessel velocity change during occlusion procedure. Arrows indicate vessel occlusion.

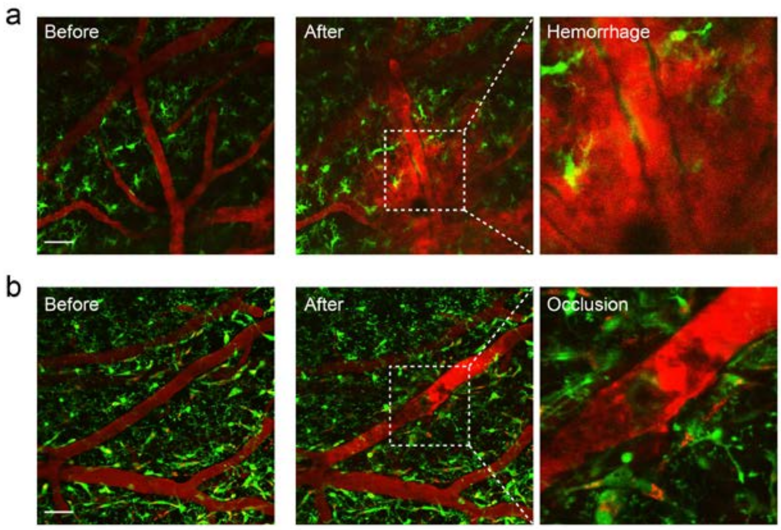

Supplementary Figure 3: Vascular hemorrhage.

a Representative image showing vascular hemorrhage via rupture of target vessel, fluorescently labeled blood plasma invading the parenchymal tissue. White square shows higher magnifications of hemorrhage image. Scale bar, 50 µm.

b Representative image showing vascular occlusion. White square shows higher magnifications of occlusion image. Scale bar, 50 µm.

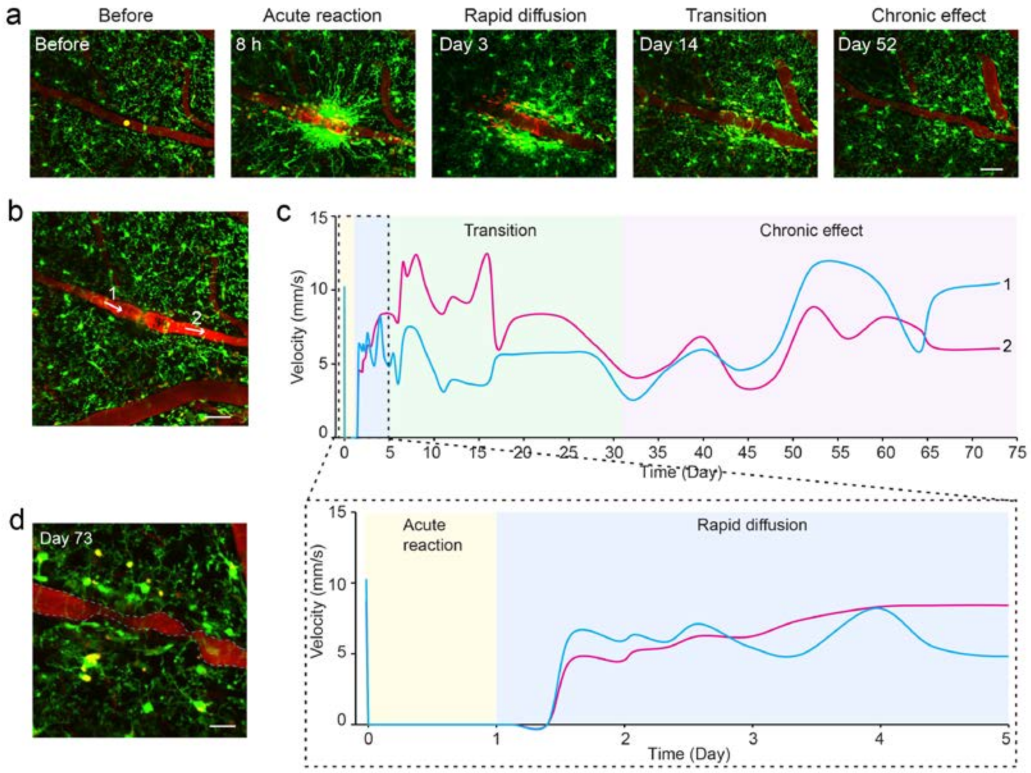

Supplementary Figure 4: Blood flow velocity dynamics.

a Representative images show microglia (green) and vessels (red) in normal physiological state (before), acute reaction phase, rapid diffusion phase, transition phase and chronic effect phase. Scale bars, 50 µm.

b Representative vessel after occlusion. White arrow indicates the blood flow direction. Scale bar, 50 µm.

c Blood flow velocity of vessel 1 (cyan) and vessel 2 (purple) shown in **b** over time. Boxed region indicates magnified view of dotted line.

d Image showing vascular malformation. Scale bar, 20 µm.

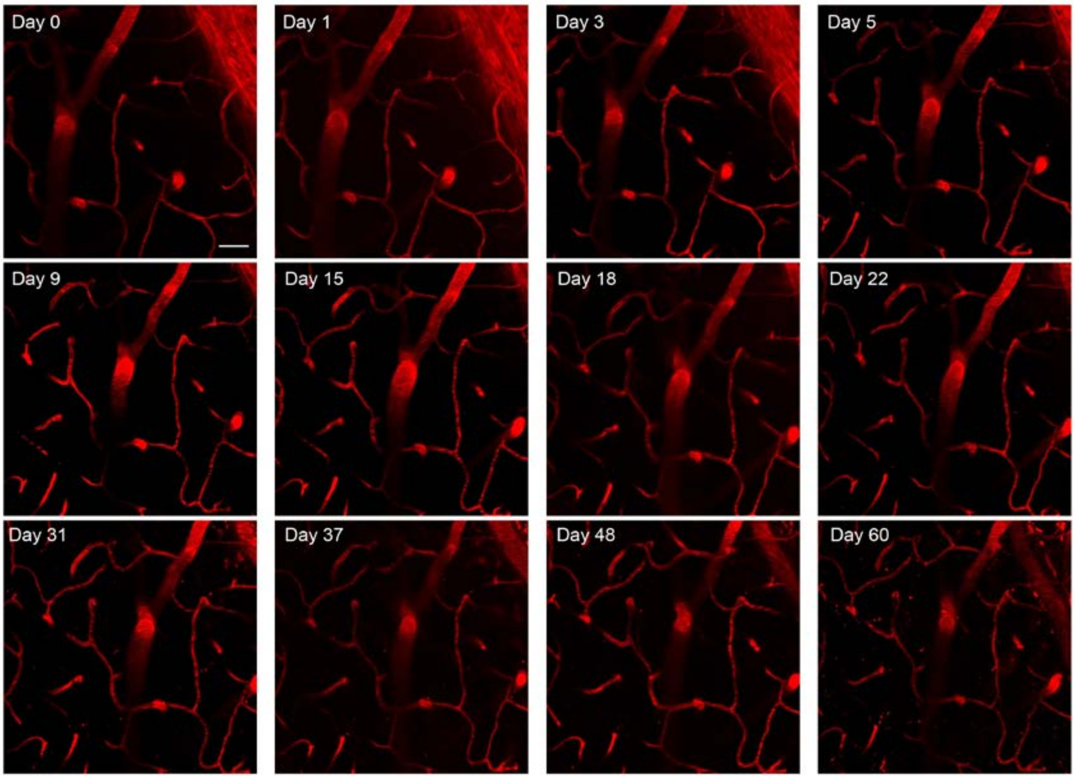

Supplementary Figure 5: Temporal sequence images of the same FOV far away from occlusion site. Scale bar, 30 μm.

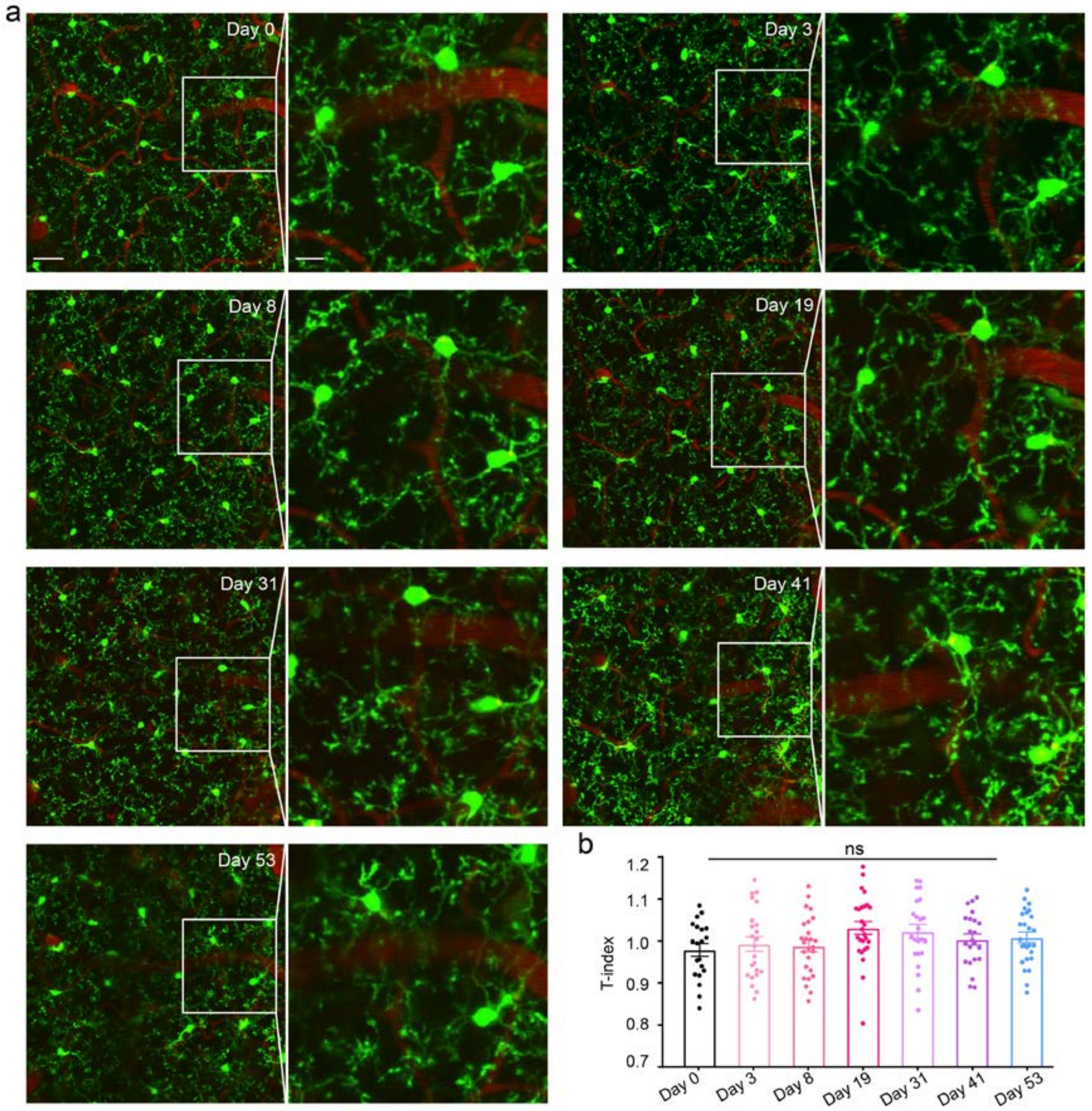

Supplementary Figure 6: Long-term *in vivo* imaging of microglia dynamics in control mouse.

a Representative images in control mouse during repeated imaging. Scale bars, 30 μm (overview) and 10 μm (magnified).

b Quantification of persistent T-index in control mouse (n = 3 mice, ns indicates no significant difference, one-way ANOVA followed by Tukey's multiple comparison). Data are presented as mean and SEM.

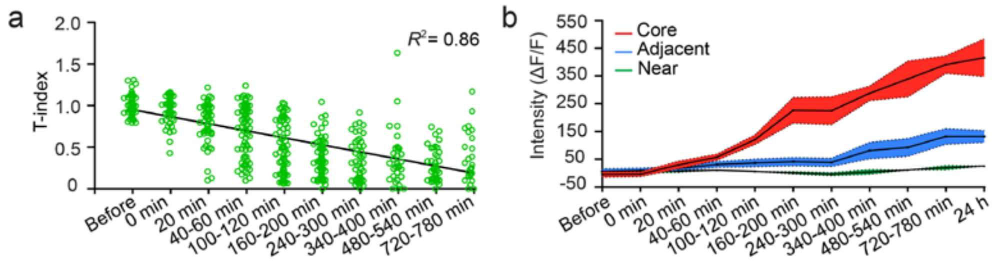

Supplementary Figure 7: Microglia dynamics in the acute reaction phase.

a Correlation between T-index and time after vessel occlusion. Pearson’s correlation test, $P < 0.001$ (n = 4 mice).

b Persistent relative intensity variance at different time points from the inner to the outer area (n = 4 mice). Data are plotted as mean (curves) ± SEM (shading around curves).

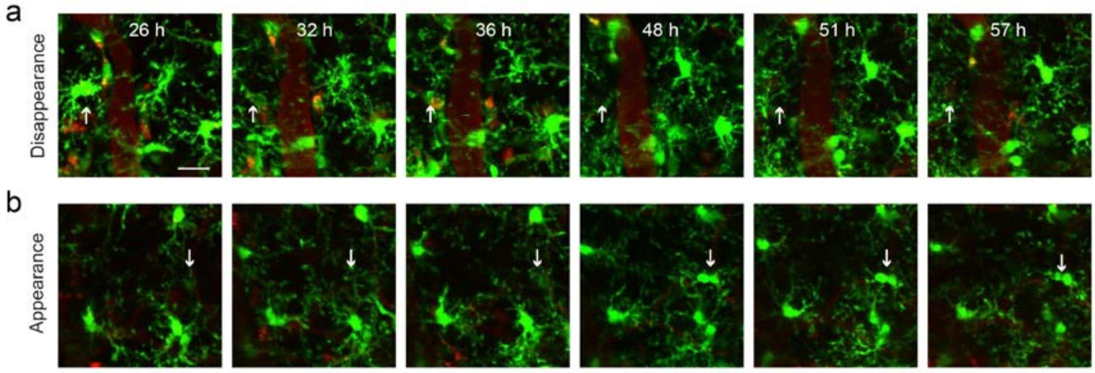

Supplementary Figure 8: Microglial translocation in the rapid diffusion phase.
a Images showing disappeared microglia during repeated imaging.
b Images showing microglial appearance during repeated imaging.
Scale bar, 20 μm.

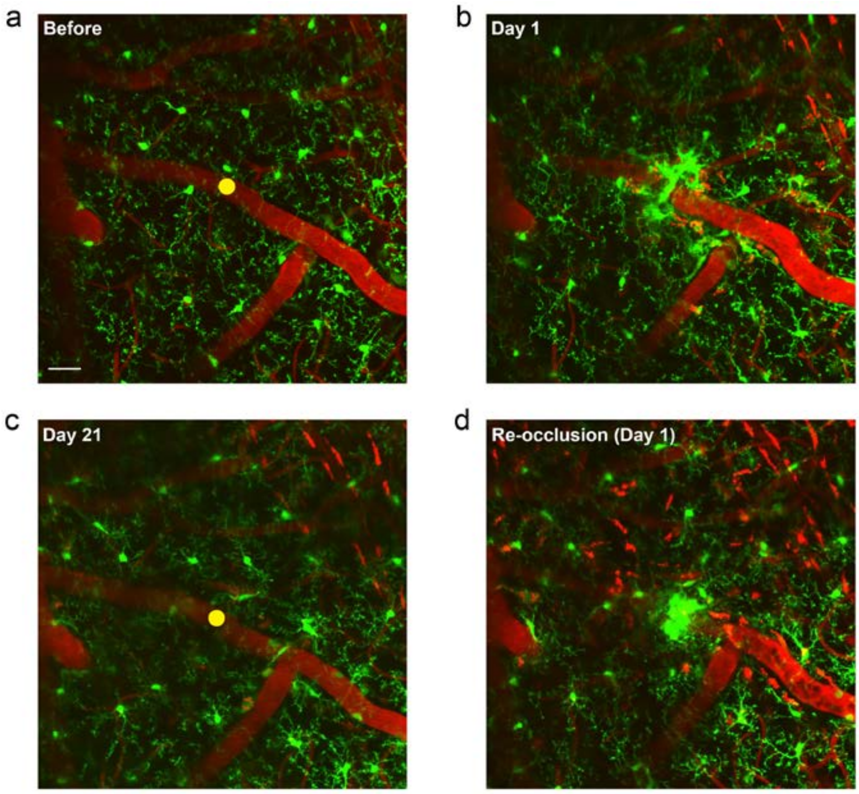

Supplementary Figure 9: Microglia in the re-occlusion area display comparable function with those prior to the occlusion.

a-b Representative image showing ramified microglia in physiological state (**a**) and microglial cluster on day 1 (**b**) after occlusion. Yellow circle indicates occlusion site.

c Microglia display ramified morphology on day 21 after occlusion. Yellow circle indicates re-occlusion site.

d In the re-occlusion area, microglia converge on the clotted site as shown in the first occlusion (**b**).

Scale bar, 30 μm.
