# Supplementary material for: Long-term microglial phase-specific dynamics during single vessel occlusion and recanalization
Source: Commun Biol. 2022 Aug 19;5:841. doi: 10.1038/s42003-022-03784-0 (PMC9391347; doi:10.1038/s42003-022-03784-0)
Supplement: Supplementary file 2 — Description of Additional Supplementary Files [file 42003_2022_3784_MOESM2_ESM.pdf]

Description of Additional Supplementary Files

File name: Supplementary Data 1

Description: Source data for Figs 1-8.

File name: Supplementary Data 2

Description: Source data for Supplementary Figs 1-9.
