# Supplementary material for: Long-term microglial phase-specific dynamics during single vessel occlusion and recanalization
Source: Commun Biol. 2022 Aug 19;5:841. doi: 10.1038/s42003-022-03784-0 (PMC9391347; doi:10.1038/s42003-022-03784-0)
Supplement: Supplementary file 5 — Reporting Summary [file 42003_2022_3784_MOESM5_ESM.pdf]

nature research

Corresponding author(s): Wei Gong

Last updated by author(s): Jul 4, 2022

Reporting Summary

Nature Research wishes to improve the reproducibility of the work that we publish. This form provides structure for consistency and transparency in reporting. For further information on Nature Research policies, see our Editorial Policies and the Editorial Policy Checklist.

Statistics

For all statistical analyses, confirm that the following items are present in the figure legend, table legend, main text, or Methods section.

n/a	Confirmed	
☐	☒	The exact sample size (n) for each experimental group/condition, given as a discrete number and unit of measurement
☐	☒	A statement on whether measurements were taken from distinct samples or whether the same sample was measured repeatedly
☐	☒	The statistical test(s) used AND whether they are one- or two-sided<br>*Only common tests should be described solely by name; describe more complex techniques in the Methods section.*
☒	☐	A description of all covariates tested
☐	☒	A description of any assumptions or corrections, such as tests of normality and adjustment for multiple comparisons
☐	☒	A full description of the statistical parameters including central tendency (e.g. means) or other basic estimates (e.g. regression coefficient) AND variation (e.g. standard deviation) or associated estimates of uncertainty (e.g. confidence intervals)
☐	☒	For null hypothesis testing, the test statistic (e.g. F, t, r) with confidence intervals, effect sizes, degrees of freedom and P value noted<br>*Give P values as exact values whenever suitable.*
☒	☐	For Bayesian analysis, information on the choice of priors and Markov chain Monte Carlo settings
☒	☐	For hierarchical and complex designs, identification of the appropriate level for tests and full reporting of outcomes
☒	☐	Estimates of effect sizes (e.g. Cohen's d, Pearson's r), indicating how they were calculated

Our web collection on statistics for biologists contains articles on many of the points above.

Software and code

Policy information about availability of computer code

Data collection	Two photon imaging: Bruker Corporation, Prairie View 5.3<br>Immunohistochemistry image: VS120, Olympus
Data analysis	ImageJ, 1.53f51<br>MATLAB 2016a ,MathWorks<br>GraphPad Prism 8

For manuscripts utilizing custom algorithms or software that are central to the research but not yet described in published literature, software must be made available to editors and reviewers. We strongly encourage code deposition in a community repository (e.g. GitHub). See the Nature Research guidelines for submitting code & software for further information.

Data

Policy information about availability of data

All manuscripts must include a data availability statement. This statement should provide the following information, where applicable:

- Accession codes, unique identifiers, or web links for publicly available datasets
- A list of figures that have associated raw data
- A description of any restrictions on data availability

All data needed to evaluate the conclusions in the paper are present in the paper and/or the Supplementary Materials. Source data are provided with this paper.

Field-specific reporting

Please select the one below that is the best fit for your research. If you are not sure, read the appropriate sections before making your selection.

☒ Life sciences ☐ Behavioural & social sciences ☐ Ecological, evolutionary & environmental sciences

For a reference copy of the document with all sections, see nature.com/documents/nr-reporting-summary-flat.pdf

Life sciences study design

All studies must disclose on these points even when the disclosure is negative.

Sample size	For all studies, 3-5 animals per experimental group were used. Samples sizes adopted in this study were sufficient for imaging.
Data exclusions	No data was excluded from analysis.
Replication	All imaging experiments were performed over multiple independent replicates. Attempts to replicate were successful once the conditions of the experiment were carefully optimized.
Randomization	All samples were randomized where appropriate for data collection and processing.
Blinding	For imaging analysis and quantification, the investigator was blinded to the groups.

Reporting for specific materials, systems and methods

We require information from authors about some types of materials, experimental systems and methods used in many studies. Here, indicate whether each material, system or method listed is relevant to your study. If you are not sure if a list item applies to your research, read the appropriate section before selecting a response.

Materials & experimental systems

n/a	Involved in the study	
☐	☒	Antibodies
☒	☐	Eukaryotic cell lines
☒	☐	Palaeontology and archaeology
☐	☒	Animals and other organisms
☒	☐	Human research participants
☒	☐	Clinical data
☒	☐	Dual use research of concern

Methods

n/a	Involved in the study	
☒	☐	ChIP-seq
☒	☐	Flow cytometry
☒	☐	MRI-based neuroimaging

Antibodies

Antibodies used	The primary antibodies used were as follows: rabbit anti-fibrinogen (1:200, Abcam, ab34269), mouse anti-MHC? (1:200, Abcam, ab23990), rabbit anti-Iba1 (1:1000, Wako, 019-19741), goat anti-Iba1 (1:500, Wako, 011-27991), rat anti-Brdu (1:200, Abcam, ab6326). The second antibodies used were as follows: donkey anti-rat 647 (1:500, Abcam, ab150155), donkey anti-rabbit 488 (1:500, Abcam, ab150073), donkey anti-goat 555 (1:500, Abcam, ab150134), donkey anti-mouse 647 (1:500, Abcam, ab150111).
Validation	All reagents are commercially available and have been validated by the respective companies.

Animals and other organisms

Policy information about studies involving animals; ARRIVE guidelines recommended for reporting animal research

Laboratory animals	C57BL/6J (B6) mice were purchased from Shanghai laboratory animal center, CX3CR1-GFP mice were purchased from Jackson Laboratories. The mice were housed in a temperature- and humidity-controlled room on a cycle of 12 h light/dark.
Wild animals	This study did not involve wild animals.
Field-collected samples	This study did not involve samples collected from the field.
Ethics oversight	Animal use and care was under the guidelines of Zhejiang University.

Note that full information on the approval of the study protocol must also be provided in the manuscript.
